# Supplementary material for: iATPSnFR2: A high-dynamic-range fluorescent sensor for monitoring intracellular ATP
Source: Proc Natl Acad Sci U S A. 2024 May 15;121(21):e2314604121. doi: 10.1073/pnas.2314604121 (PMC11126915; doi:10.1073/pnas.2314604121)
Supplement: Supplementary file 1 — Appendix 01 (PDF) [file pnas.2314604121.sapp.pdf]

Supplementary Figures

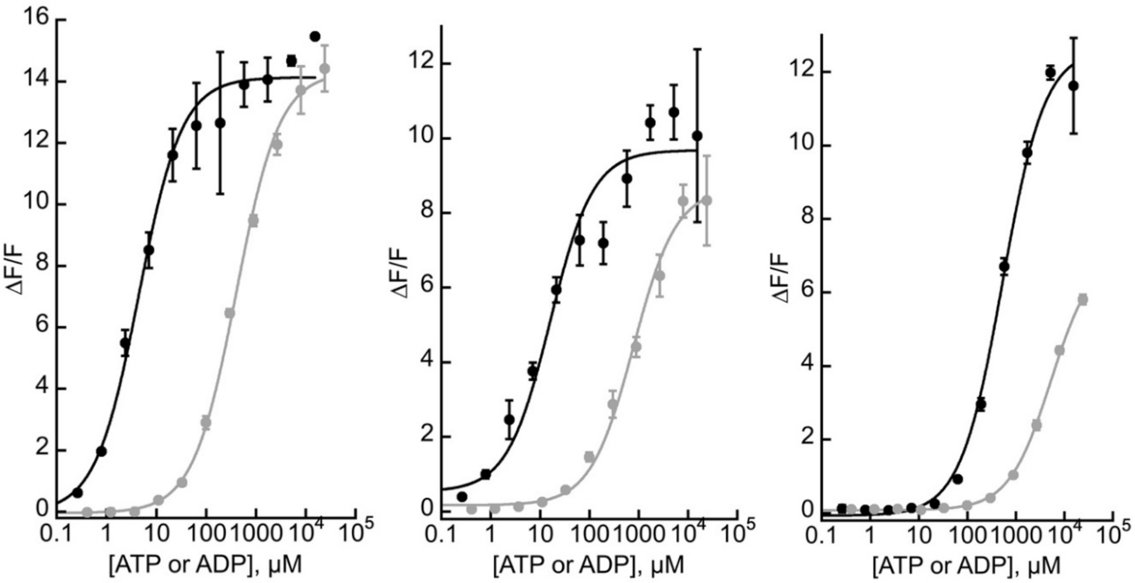

Supplementary Figure 1a. _In vitro_ characterization: ATP & ADP affinity. Three variants of iATPSnFR2.HaloTag-JFX650 titrated with ATP (black) and ADP (grey), head-to-head. iATPSnFR2.S29W.A95K (left); iATPSnFR2.A95K (center); iATPSnFR2.A95A.A119L (right). Binding curve fits, in μM, for ATP and ADP are S29W.A95K: 4 μM, 400 μM. A95K: 16 μM, 780 μM. A95A.A119L: 530 μM, 5300 μM. Error bars are s.d. of three technical replicates.

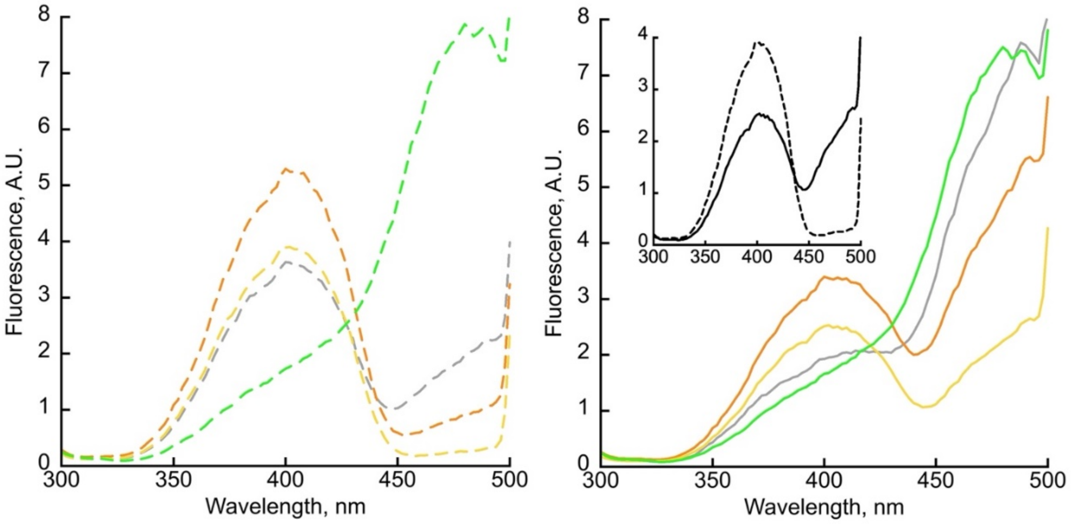

Supplementary Figure 1b. <u>*In vitro* characterization: excitation spectra.</u> iATPSnFR2.S29W (grey), iATPSnFR2.A95K (orange), iATPSnFR2.A95A.A119L (yellow), and cpSFGFP (green) in the absence of ATP (left, dashed lines) and in the presence of 7 mM ATP (right, solid lines). Emission observed at 515 nm (5 nm bandpass). Excitation scanned with 5 nm bandpass. Inset shows A95A.A119L variant spectra ± ATP to indicate the isosbestic point. Spectra are single data collections.

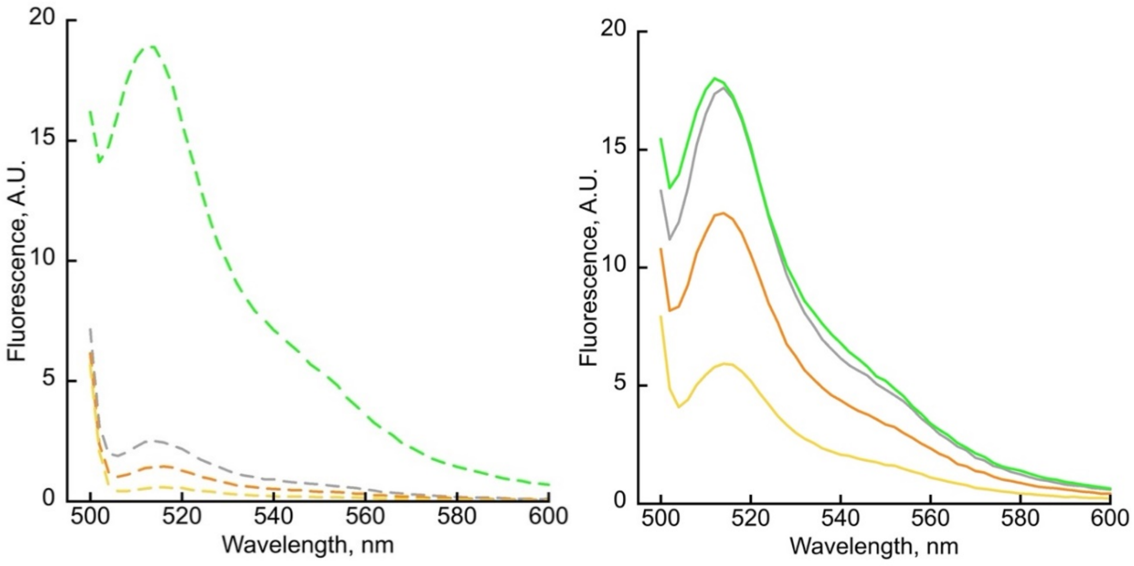

Supplementary Figure 1c. <u>*In vitro* characterization: emission spectra.</u> iATPSnFR2.S29W.A95K (grey), iATPSnFR2.A95K (orange), iATPSnFR2.A95A.A119L (yellow), and cpSFGFP (green) in the absence of ATP (left, dashed lines) and in the presence of 7 mM ATP (right, solid lines). Excitation at 485 nm (5 nm bandpass). Emission scanned with 5 nm bandpass. Spectra are single data collections.

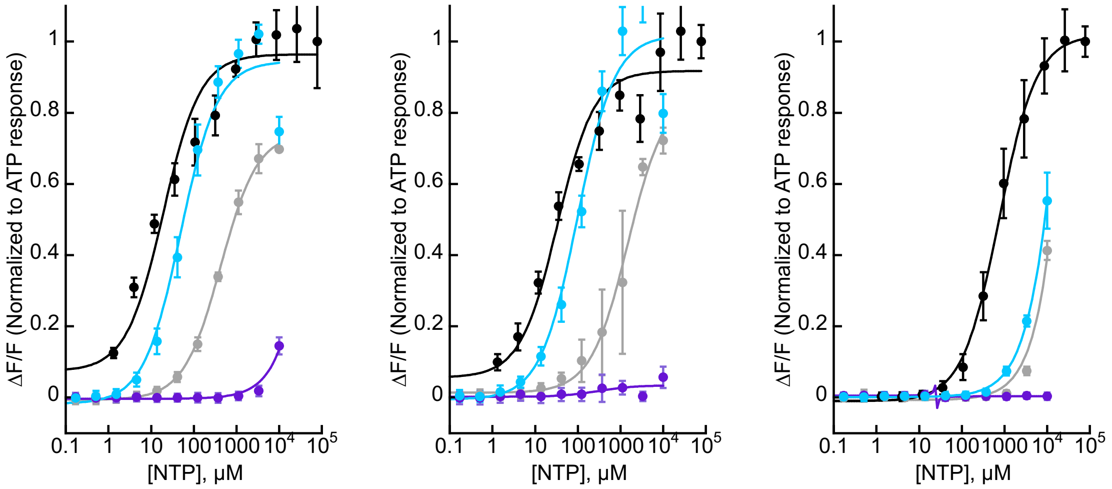

Supplementary Figure 1d. *In vitro* characterization: binding specificity. iATPSnFR2 affinity for ATP and other nucleoside triphosphates. Three variants of iATPSnFR2.HaloTag-JFX650 titrated with ATP and other NTPs, head-to-head. S29W.A95K (left); A95K (center); A95A.A119L (right). ATP (black), CTP (cyan), GTP (grey), TTP (purple). Binding curve fits, in μM, for ATP, CTP, GTP are S29W.A95K: 20 μM, 50 μM, 430 μM. A95K: 30 μM, 100 μM, 1500 μM. A95A.A119L: 750 μM, ~10 mM, ~10 mM. Error bars are s.d. of three technical replicates.

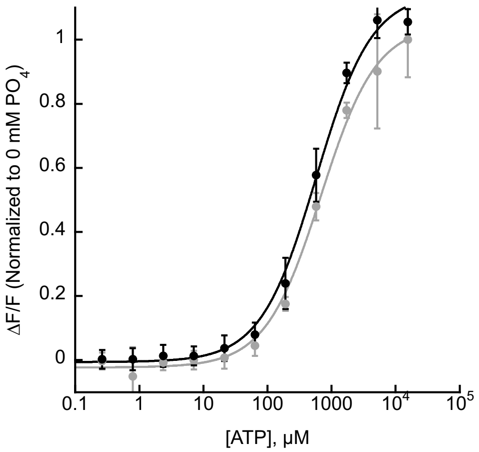

Supplementary Figure 1e. *In vitro* characterization: inorganic phosphate dependence. The iATPSnFR2.A95A.A119L.HaloTag-JFX650 sensor is negligibly affected by inorganic phosphate. Head-to-head titration of ATP without added phosphate (black) and with 20 mM PO_4 (grey) (diluted from 1 M $Na(PO_4)$ stock, pH 7). Error bars are s.d. of three technical replicates.

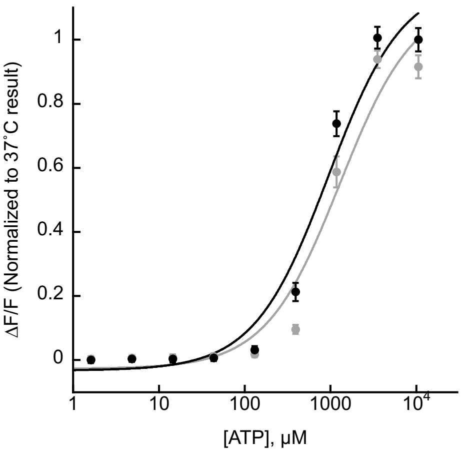

Supplementary Figure 1f. *In vitro* characterization: temperature dependence. ATP titration of iATPSnFR2.A95A.A119L variant at 25˚C (grey) and 37˚C (black). Error bars are s.d. of three technical replicates. Fluorescence was measured in a Cytation 5 plate reader, which has relatively rapid heating.

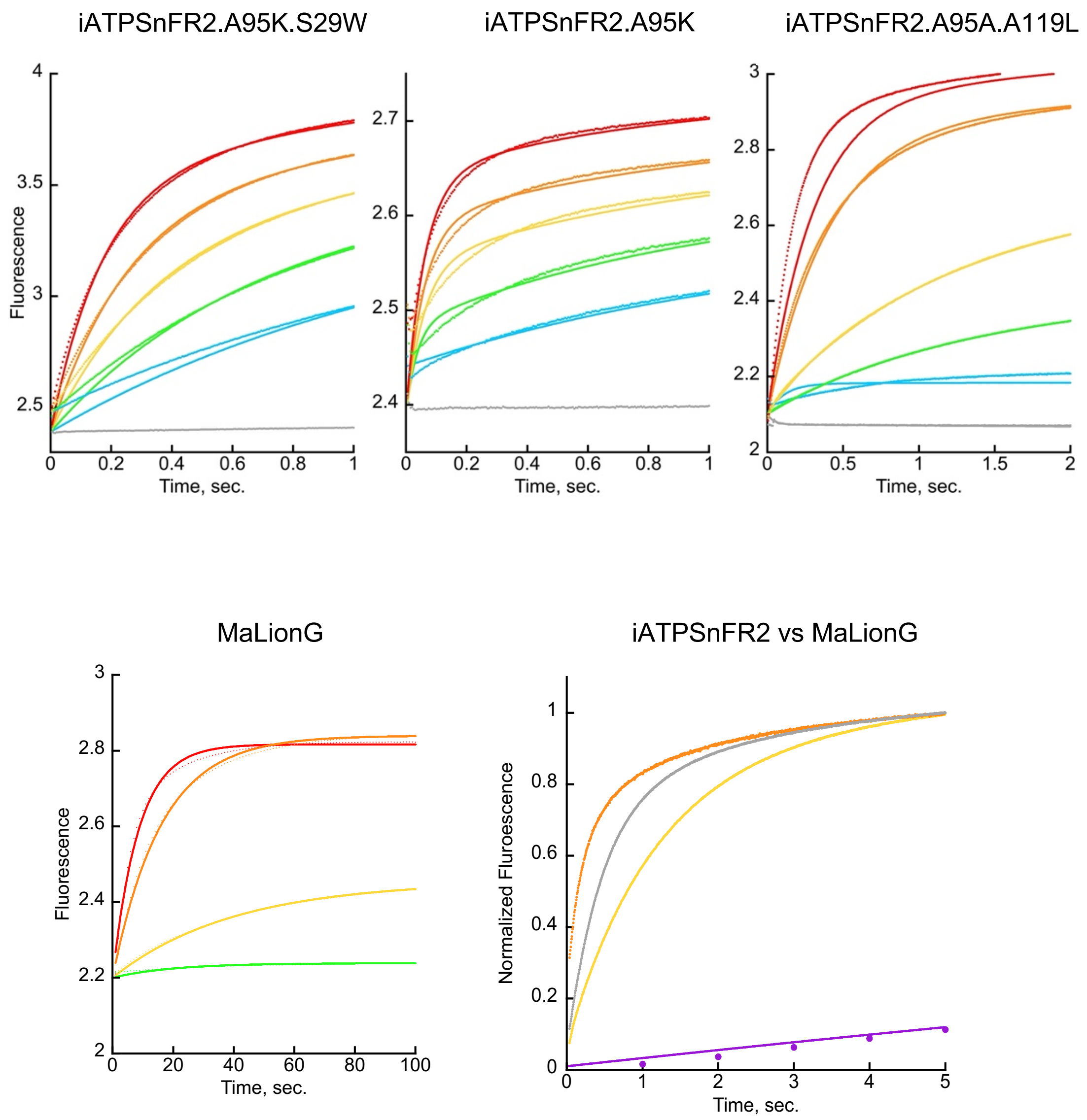

Supplementary Figure 1g. <u>*In vitro* characterization: kinetics</u>. TOP: The three different variants of iATPSnFR2 bind ATP within a one or two seconds of rapid mixing. S29W.A95K (top left) and A95K (top center) bind ATP faster than A95A.A119L (top right). The stopped flow fluorescence kinetics data required a double exponential fit, indicating a more complicated, three-state mechanism of binding and fluorescence change. Top panels are the sensor with a C-terminal fusion to HaloTag-JFX650. BOTTOM: MaLionG binds ATP at a much slower rate (bottom left). Final concentrations of ATP are: 10000 (red), 3333 (orange), 1111 (yellow), 370 (green), 123 µM (blue). Fluorescence data collected on an Applied Photophysics SX-20 stopped-flow apparatus with 490 nm LED excitation and 525 nm long pass filter. Data points are average of 5 technical replicates. Curve fit (solid line) is a single exponential. Time scale comparison of all four sensors (bottom right): time course of fluorescence increase upon mixing 2.222 mM ATP (final concentration = 1.111 mM) with the three affinity variants of iATPSnFR2 (yellow: A95A.A119L, orange: A95K, grey: A95K.S29W) or MaLionG (purple). Responses are normalized to initial (0) and final (1) fluorescence values. Data points are average of five technical replicates.

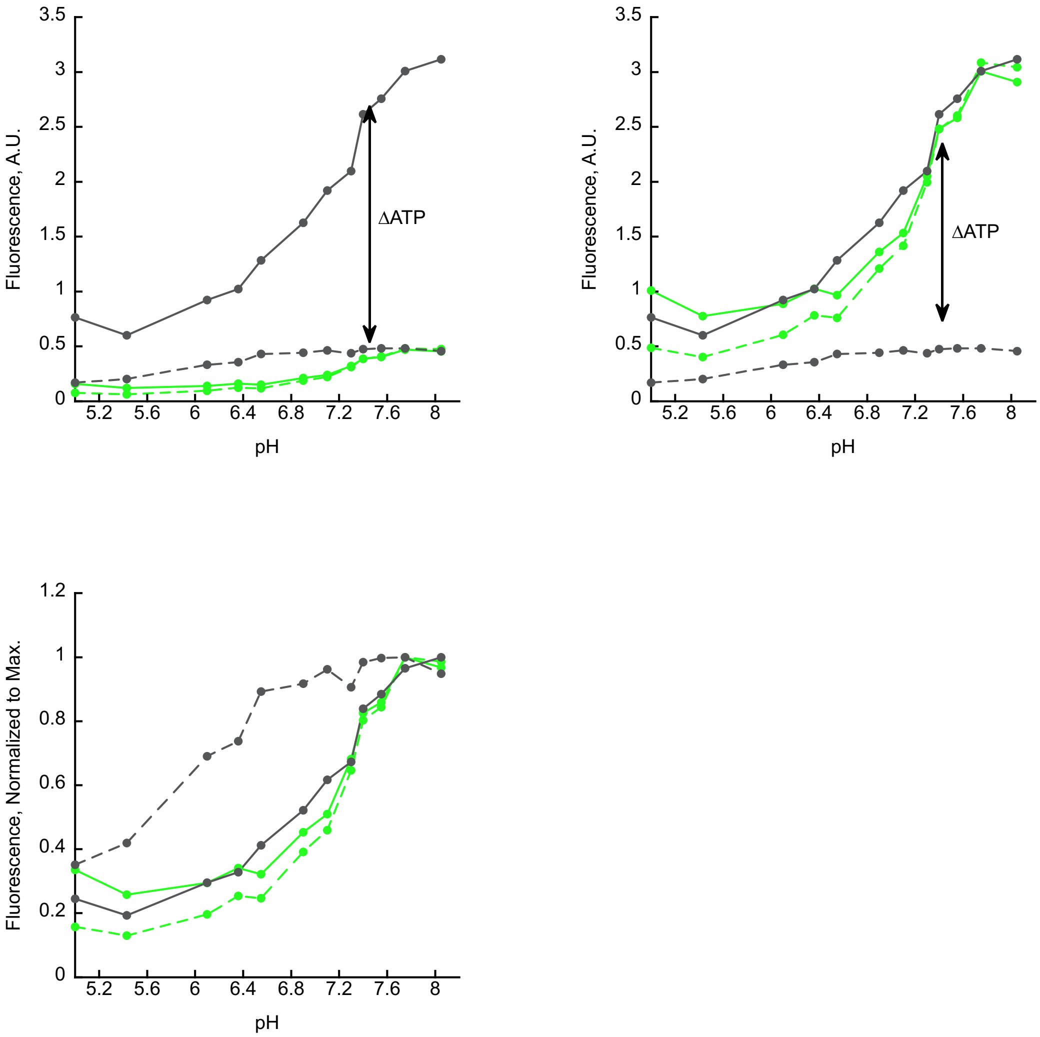

Supplementary Figure 1h. *In vitro* characterization:pH dependence. iATPSnFR2.A95A.A119L.HaloTag and cpSFGFP were diluted to 0.2 μM in Mammalian Cell Imaging Buffer at variable pH. Fluorescence was measured at Ex/Em 485 nm / 535 nm (20 nm bandpass) or Ex/Em 600 nm / 630 nm (10 nm bandpass) without ATP (dashed lines). Then ATP was added to 2.5 mM from a concentrated stock and fluorescence was remeasured (solid lines). Each plot is a different presentation of the same data. In the top left panel, the fluorescence of cpSFGFP has been adjusted to approximately match that of ligand-free state of the sensor at pH 7.4. In the top right panel, the fluorescence of cpSFGFP has been adjusted to match that of the ATP-bound state of the sensor at pH 7.4. In the lower left plot, each data set has been adjusted so that its maximum value is 1. (Black, iATPSnFR2 green channel; green, cpSFGFP green signal). Data points are average of three technical replicates.

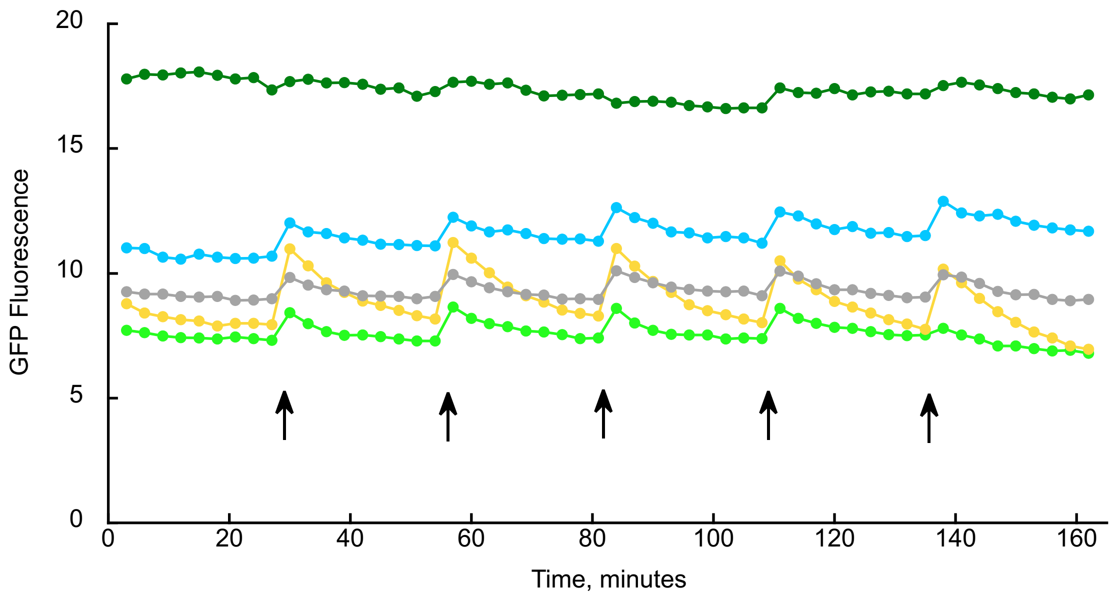

Supplementary Figure 1i. Artefacts of fluorescence imaging in Cytation5 multi-well plate reader. COS7 cells transfected with iATPSnFR2 variants (yellow: A95A.A119L, grey: S29W.A95K), which contain cpSFGFP, or cpSFGFP itself (light green), or MaLionG (blue), which contains split GFP. HEPES buffer containing 10 mM glucose was removed and added back (not exchanged for fresh buffer) at the points indicated by arrows. eGFP (dark green) does not appreciably change fluorescence in this assay. Data points are the average fluorescence of ROIs determined by thresholding in ImageJ. Number of cells analyzed for each sample are: GFP 50, cpSFGFP 31, AL 31, S29W 37, MaLionG 13.

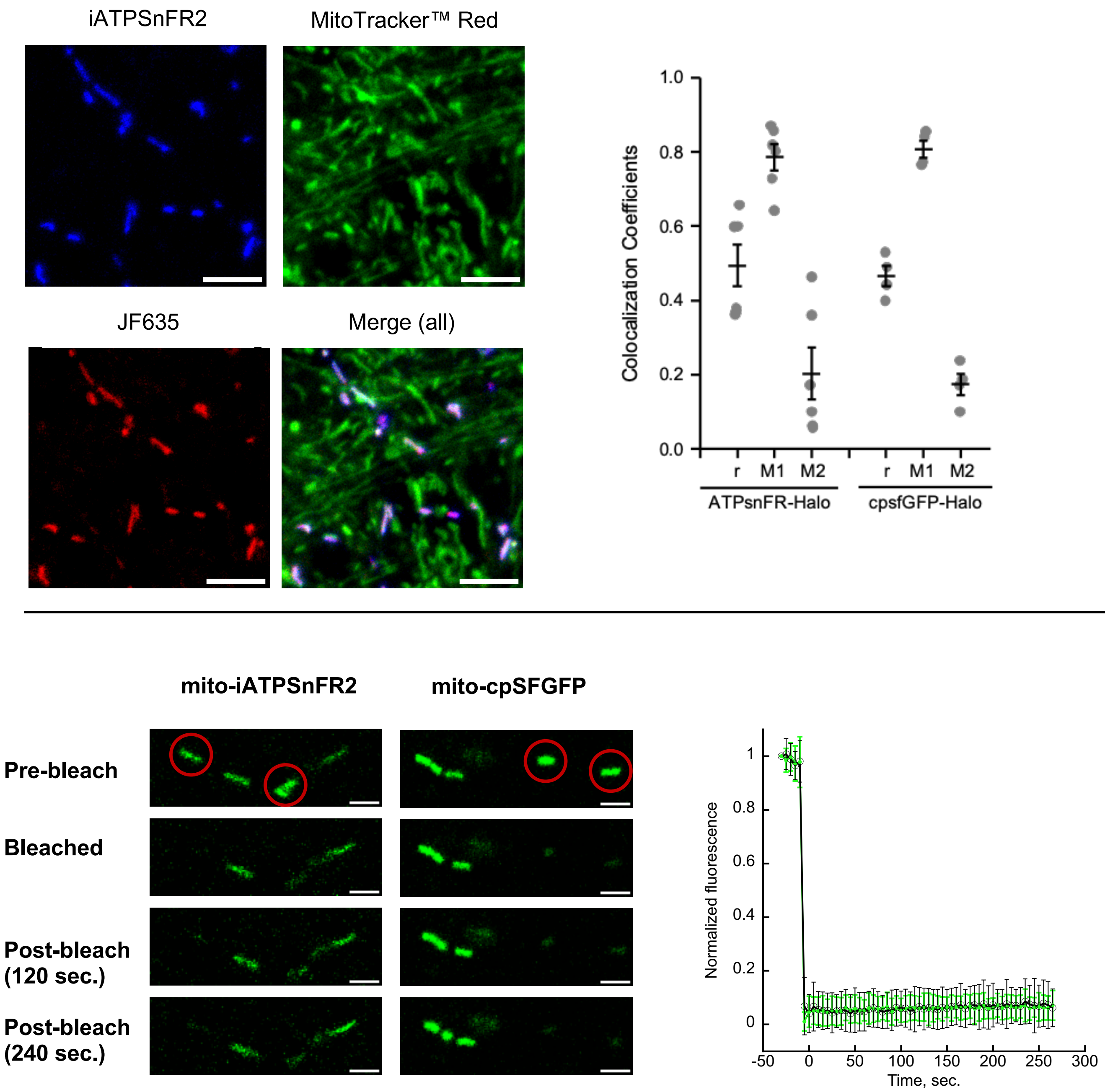

Supplementary Figure 2a. Validation of mitochondrial targeting of mito-iATPSnFR2.HaloTag in neuronal culture. TOP: Image of hippocampal neuronal culture transfected with mito-iATPSnFR2.A95A.A119L.HaloTag and labeled with JF635-HaloTagLigand (200 nM) and MitoTracker™ Red (100 nM). Upper left (blue channel): green fluorescence from iATPSnFR2; upper right (green channel): red fluorescence from MitoTracker™ Red; lower left (red channel): far-red fluorescence from HaloTag-JF635; lower right: merge of all three channels. Right: Co-localization coefficients of iATPSnFR2-HaloTag with MitoTracker™ Red. M1: Fraction of HaloTag-JF635 overlap with MitoTracker™ Red; M2: Fraction of MitoTracker™ Red overlap with HaloTag-JF635; r: Pearson's coefficient. BOTTOM: Fluorescence recovery after photobleaching. Left: Representative images of mitochondrially targeted iATPSnFR2 or cpSFGFP before bleaching and at timepoints afterwards. Scale bar 2 μm. Right: quantification of recovery from 15 ROIs.

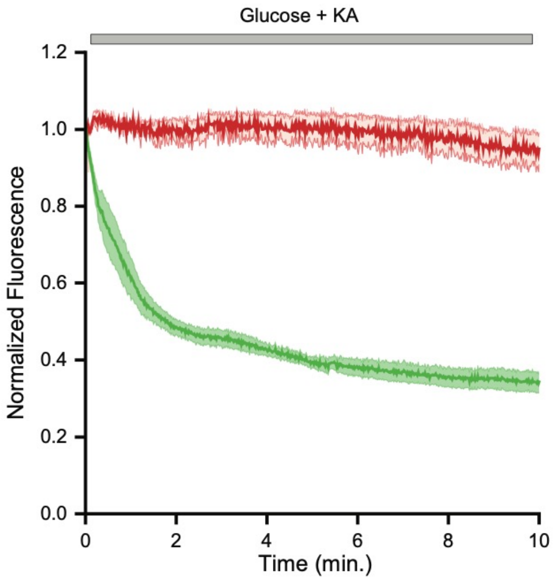

Supplementary Figure 2b. Validation of mito-iATPSnFR2.HaloTag in neuronal culture. Fluorescence traces of both green (iATPSnFR2) and red (HaloTag-JF635) channels in neurons expressing mito-iATPSnFR2.A95A.A119L.HaloTag in mitochondria during perfusion with 5 mM glucose and 10 µM KA. Fluorescence traces from 4 neurons (mean ± SEM) with each neuron providing signals form 40-50 axonal mitochondria are shown.

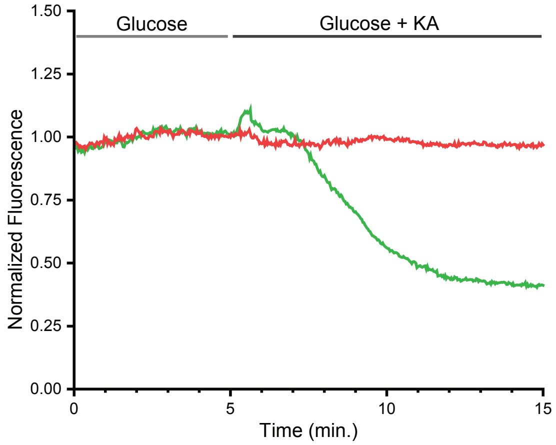

Supplementary Figure 2c. Validation of mito-iATPSnFR2.miRFP670nano3 in neuronal culture. Average fluorescence traces of both green (iATPSnFR2) and red (mIRFP670nano3) channels from 4 neurons expressing mito-iATPSnFR2.A95A.A119L.mIRFP670nano3 during perfusion with 5 mM glucose followed by 5 mM glucose with 10 µM KA starting at 5th minute.

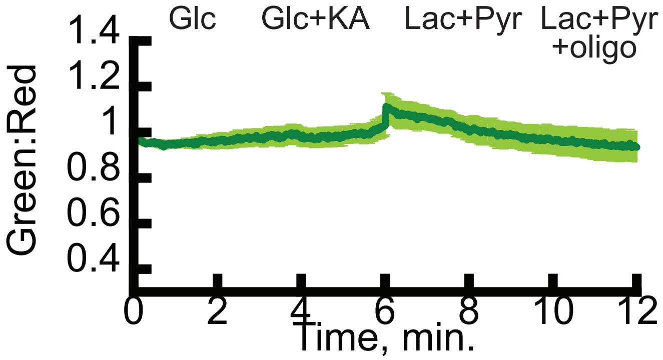

Supplementary Figure 2d. Response of copSFGFP in matrix of axonal mitochondria to glycolytic and mitochondrial ATP synthase inhibitors. cpSFGFP.HaloTag-JF635 targeted to mitochondria with 4x-COX8 signal sequence was imaged and treated identically as iATPSnFR2 was in Fig. 2. Data is represented as mean of 300 mitochondria across 6 cells ± S.E.M.

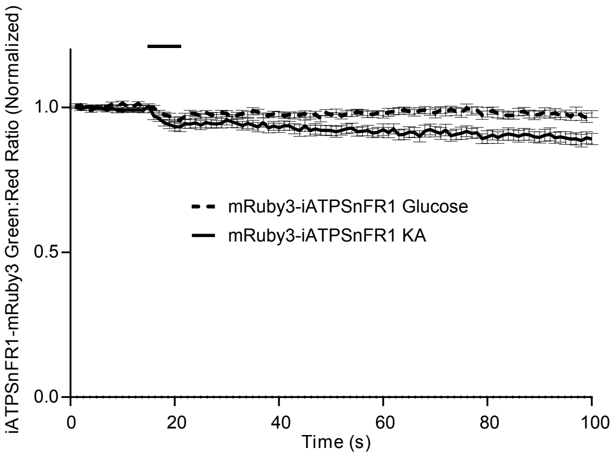

Supplementary Figure 3a. Fluorescence response of iATPSnFR1 in neurons to electrical stimulation (black bar). Stimulation in 5 mM glucose (dashed) or under glycolytic block containing 10 µM KA (solid). Average of 18 cells ± SEM.

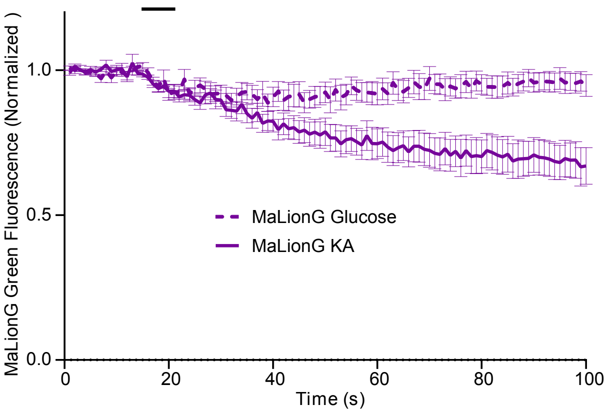

Supplementary Figure 3b. Fluorescence response of MaLionG in neurons to electrical stimulation (black bar). Stimulation in 5 mM glucose (dashed) or under glycolytic block containing 10 µM KA (solid). Average of 9 cells ± SEM.

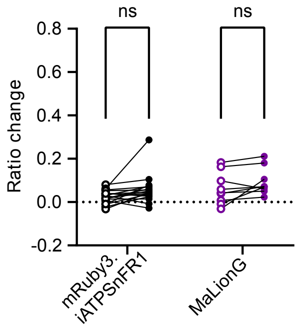

Supplementary Figure 3c. Statistical analysis of fluorescence response of iATPSnFR1 and MaLionG in neurons to electrical stimulation. Fluorescence change at the end of the stimulation period. Open circles: in buffer containing 5 mM glucose; closed circles: in buffer containing 5 mM glucose + 10 µM KA. $^{ns}p > 0.05$ paired t-test.

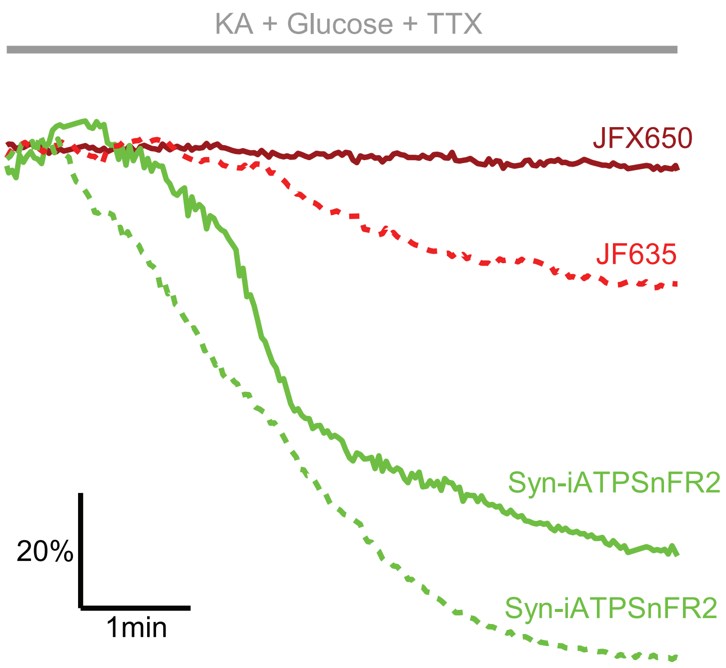

Supplementary Figure 4a. Validation of Syn-iATPSnFR2.HaloTag in axonal boutons. Fluorescence traces of both green (iATPSnFR2) and red (HaloTag-JF635 or HaloTag-JFX650) channels in neurons expressing (synapto)-iATPSnFR2.A95A.A119L.HaloTag on the cytosol-facing surface of synaptically targeted vesicles during perfusion with 5 mM glucose and 10 µM KA. Solid lines: (synapto)-iATPSnFR2.A95A.A119L.HaloTag-JFX650. Dashed lines: (synapto)-iATPSnFR2.A95A.A119L.HaloTag-JF635.

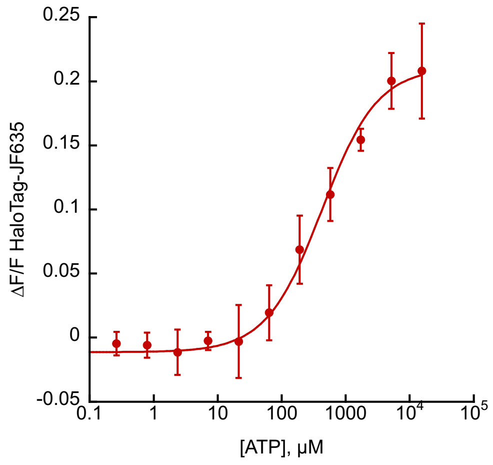

Supplementary Figure 4b. *In vitro* titration of HaloTag-JF635 with ATP. Fluorescence of purified HaloTag-JF635 (0.2 uM in Mammalian Cell Imaging Buffer) was measured (Ex 625 nm, Em 670 nm, 20 nm bandpass) with varying concentration of ATP.

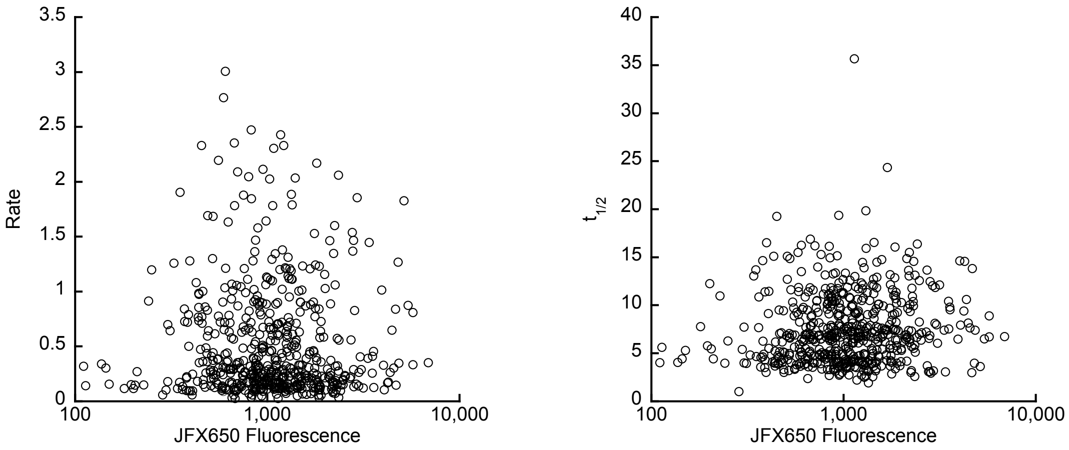

Supplementary Figure 4c. Fluorescence decay in boutons is not correlated to sensor expression. Scatter plot of fluorescence decay rate (left) or $t_{1/2}$ (right) and sensor expression (as determined by brightness of HaloTag-JFX650).

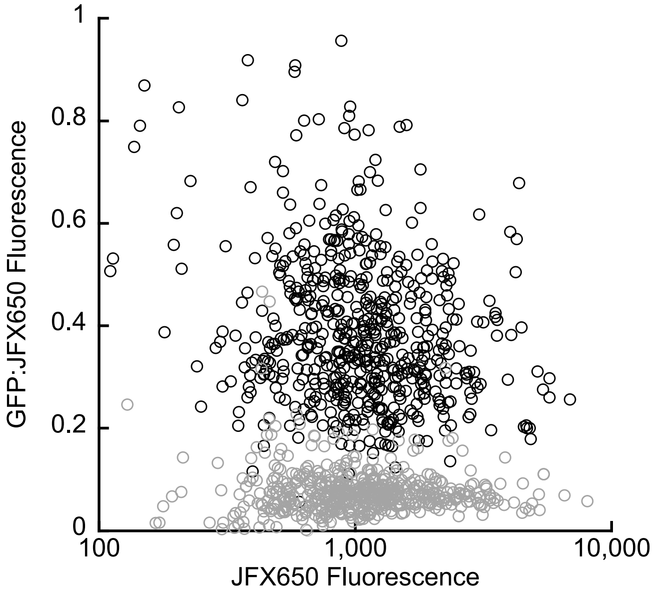

Supplementary Figure 4d. Apparent ATP concentration in boutons is not correlated with sensor expression. Scatter plot of iATPSnFR2:HaloTag-JFX650 fluorescence (proxy for ATP concentration) vs JFX650 fluorescence (proxy for sensor expression) for all boutons in Fig. 4. Dark circles: boutons before KA treatment; grey circles: boutons after KA treatment.
